# Characteristics of Antisense Transcript Promoters and the Regulation of Their Activity

**DOI:** 10.3390/ijms17010009

**Published:** 2015-12-23

**Authors:** Shudai Lin, Li Zhang, Wen Luo, Xiquan Zhang

**Affiliations:** 1 Department of Animal Genetics, Breeding and Reproduction, College of Animal Science, South China Agricultural University, Guangzhou 510642, China; shudailin@stu.scau.edu.cn (S.L.); zhangli761101@163.com (L.Z.); lw729@stu.scau.edu.cn (W.L.); 2 Guangdong Provincial Key Lab of Agro-Animal Genomics and Molecular Breeding, Guangzhou 510642, China; 3 Key Lab of Chicken Genetics, Breeding and Reproduction, Ministry of Agriculture, Guangzhou 510642, China; 4 Agricultural College, Guangdong Ocean University, Zhanjiang 524088, China

**Keywords:** antisense promoter, location, characteristics, activity, influencing factors

## Abstract

Recently, an increasing number of studies on natural antisense transcripts have been reported, especially regarding their classification, temporal and spatial expression patterns, regulatory functions and mechanisms. It is well established that natural antisense transcripts are produced from the strand opposite to the strand encoding a protein. Despite the pivotal roles of natural antisense transcripts in regulating the expression of target genes, the transcriptional mechanisms initiated by antisense promoters (ASPs) remain unknown. To date, nearly all of the studies conducted on this topic have focused on the ASP of a single gene of interest, whereas no study has systematically analyzed the locations of ASPs in the genome, ASP activity, or factors influencing this activity. This review focuses on elaborating on and summarizing the characteristics of ASPs to extend our knowledge about the mechanisms of antisense transcript initiation.

## 1. Introduction

Natural antisense transcripts comprising both coding and non-coding regulatory RNAs are widespread in the mammalian transcriptome. Indeed, it is estimated that at least 22%–40% of genes have an antisense partner [1,2,3]. These antisense transcripts contain sequences that are partially or completely complementary to sense transcripts and can interact with their sense RNAs through these complementary regions [4,5].

Previous studies have revealed many characteristics of natural antisense transcripts, including their classification [6,7,8,9], spatial and temporal expression profiles [10,11,12], regulatory functions and molecular mechanisms [10,13,14,15,16,17,18,19,20,21,22]. Natural antisense transcripts are involved in early embryonic developmental processes, such as cell proliferation [10,23], and cell migration and sprouting [10], as well as in healthy growth and disease. Through direct interaction with sense transcripts or indirect interaction with other targets, natural antisense transcripts have a widespread impact on conventional gene expression at the DNA and genome, transcriptional, post-transcriptional and translational levels [20]. The reported models of action include transcriptional collision [24], DNA methylation, histone modification [25], mRNA stability [10,26,27], endogenous siRNA [28], and mRNA splicing, editing and translation [4,29]. Chromatin modification as a result of ectopic expression of natural antisense transcripts has even been found to be dysregulated in human disease [5,13,23,25,30]. These previous studies indicate that antisense transcripts are tissue specific and may be regulated by promoters in the same manner as sense transcripts.

However, the transcription mechanism of antisense transcripts remains poorly understood. Similar to sense transcription driven by a promoter, antisense transcripts are controlled by antisense promoters (ASPs) that can be recognized by transcription factors (TFs) to form a transcription initiation complex [31]. Therefore, further antisense transcript studies may focus on identifying the regulation of target gene expression by new functional natural antisense transcripts in specific tissues or cells, simultaneously exploring the relationship between certain critical diseases and the regulation mechanisms of natural antisense transcripts themselves. For example, expression of the lymphoid enhancer factor 1 (*LEF1*) gene is attenuated by a natural antisense transcript that is regulated by the balance between its spliced and unspliced forms [32]. Greater insight through biomedical research into the regulation mechanisms of natural antisense transcripts may provide novel diagnostic labels and drug targets in clinical applications.

## 2. Location of Antisense Promoters (ASPs)

**Figure 1:**
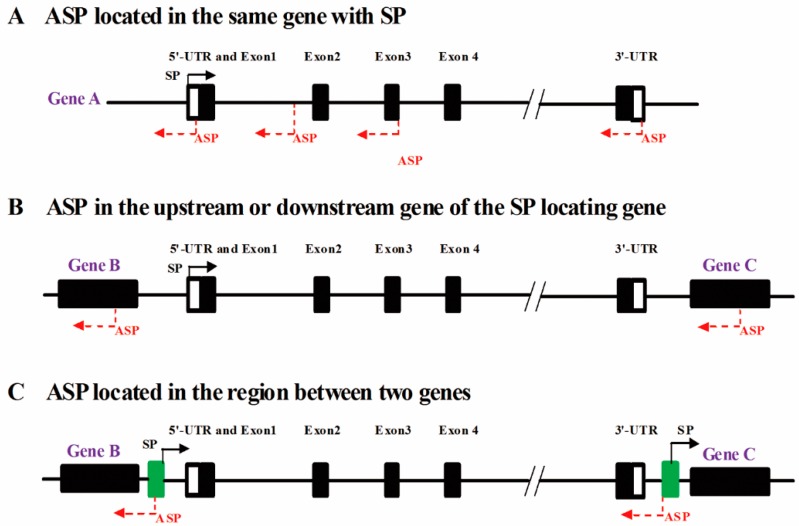
Characteristics of the locations of antisense promoters (ASPs). When an ASP (red reverse dotted arrow) and the sense promoter (SP) (black solid arrow) are located in the same gene (**A**); the ASP can be located at any position in the 5′-UTR, exons and introns or 3′-UTR. ASPs can also be located in genes upstream or downstream (gene B or C, respectively) of the SP gene (**B**); or an ASP may be located between two genes, with SP in the same region (green rectangle) (**C**). In the figure, “/ /” indicates other exons and introns.

Identification of the positions of ASPs can help in clarifying their potential regulatory functions and analyzing the transcription start sites (TSSs) and transcriptional mechanisms of natural antisense transcripts [33]. ASPs are generally located on the strand opposite from coding genes [33,34]. The locations of ASPs can be divided into three types relative to the locations of the sense transcript promoters (SPs): in the same gene as the SP (Figure 1A), in genes upstream or downstream of the SP (Figure 1B), or in intergenic regions (Figure 1C). When an ASP is located in the same gene as the SP, it can be present in the 5′-UTR, exons, introns or 3′-UTR of the gene (Figure 1A).

### 2.1. Both ASPs and Sense Promoters (SPs) Can Be Located in the Same Gene

#### 2.1.1. ASPs and SPs Are Located in the 5′-UTR of the Same Gene

**Figure 2:**
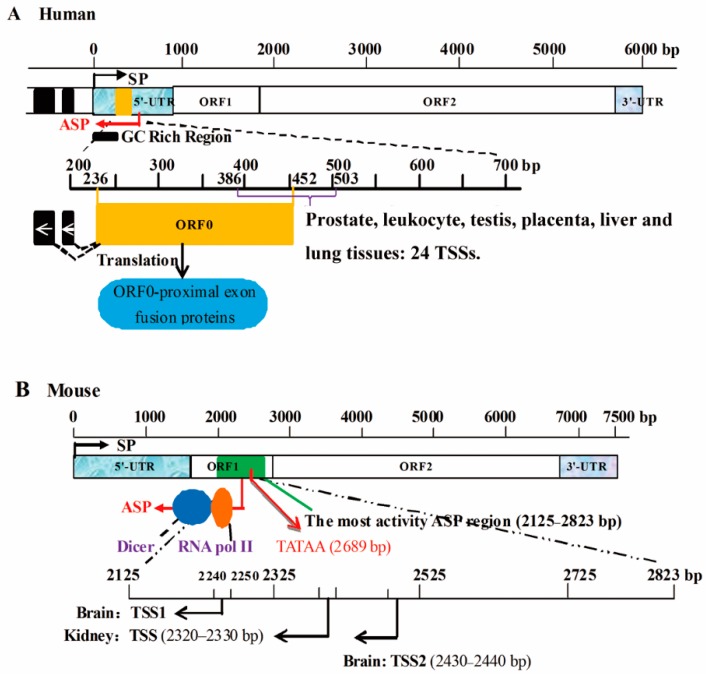
Schematic representation of the position of L1 ASPs in humans and mice. (**A**) The L1 5′-UTR (human) harbors an ASP (red reverse arrow) and a SP (black arrow) [36,37]; the mouse L1 ASP (red arrow showed in (**B**) is within ORF1 [37]. The G-C base rich (GC-rich) region (0–485 bp, black box) plays a role in the activity of the L1 ASP. There are 24 TSSs (transcription start sites) in purple arrow mapped to the opposite strand of the L1 5′-UTR. Interestingly, the L1 ASP functions as an alternative promoter, generating not only antisense transcripts, but also chimeric mRNAs [36]. ORF0 (yellow rectangle), a primate-specific open reading frame-0 located in 236–452 bp, not only contributes to the generation of ORF0-proximal exon fusion proteins but also enhances L1 mobility and there are 24 TSSs of various tissues located in 386–503 bp (purple bracket) [25]. The wright arrows means the transcriptional direction of ORF0, and the black dotted line and connected black boxes represent its transcript alternative splicing; (**B**) The mouse L1 ASP is located in ORF1, which includes a TATAA sequence at 2689 bp (orange arrow) and the ASP region with the highest activity from 2125 to 2823 bp (green frame) [37]. It has been demonstrated that RNA polymerase II (orange circle) and Dicer (blue circle) participate in the L1 ASP transcriptional initiation complex, with the latter playing a modest role in limiting native L1 retrotransposition. There are also different TSSs located in the L1 ASP, such as brain tissue-specific TSSs located at 2240–2250 bp and 2430–2440 bp (black reverse arrow); a kidney-specific TSS is located at 2320–2330 bp (black reverse arrow). The difference between the L1 ASPs of humans and mice is due to evolutionary events [3].

SPs are usually located in the 5′-UTR of a gene, though ASPs can be simultaneously present in the gene’s 5′-UTR. For example, the V1 promoter, the SP of the chicken growth hormone receptor (*GHR*) gene is located in the 5′-UTR of the gene [35]. Additionally, both the SP and ASP of human long interspersed elements (L1s) are located in the 5′-UTR [3,36,37], and the SP and ASP can compete, leading to six alternative splicing patterns of L1 5′-UTR transcripts (Figure 2) [36,38]. Recently, it has been reported that an L1 primate-specific open reading frame-0 (ORF0), can contribute to the generation of ORF0-proximal exon fusion proteins and enhance L1 mobility (Figure 2) [25]. In addition, the human tryptophan hydroxylase-2 (*hTPH2*) 5′-UTR harbors a bidirectional promoter; the ASP is much more active than the SP, but both are cell line dependent, with the highest activities observed in Human Embryonic Kidney 293T cell line containing the SV40 Large T-antigen (HEK-293T) and the lowest in a human neuroblastoma cell line (SK-N-MC) [39]. Bidirectional promoters can transcribe both sense transcripts and antisense transcripts, and their TSSs may be located more than 1 kb apart [7,40,41]. Thus, it is clear that an ASP and SP can exist simultaneously in a 5′-UTR.

SPs and ASPs compete for a portion or all of the elements in the 5′-UTR region and generate typical head-to-head transcripts. Finocchiaro *et al.* [33] discovered 7072 sense/antisense transcript pairs in the human genome, and found that 76% exhibit a head-to-head formation, whereas the rest are intragenic pairs. Remarkably, 58% of antisense transcripts begin from a 500 bp region upstream of the TSS of protein-coding genes, indicating that both sense and antisense transcripts can be produced from the same promoter element. This phenomenon is characterized by a specialized transcriptional control mechanism that is directly coupled to relaxed bidirectional transcription [42]. Therefore, bidirectional transcription may involve many specific TF binding sites (TFBSs) controlling different directions of SPs and ASPs in an orderly manner.

#### 2.1.2. ASPs Are Located in Exons

ASPs may be located in the exons of a gene when SPs are located in the 5′-UTR. For example, the *Dictyostelium discoideum* prespore gene *EB4-PSV* ASP is located in exon 3, driving an antisense transcript that terminates in the SP region [43]. Unlike the human L1 ASP located in the L1 5′-UTR, the rat L1-ASP is located in open reading frame-1 (ORF1) (Figure 2) [37], and the L1-ASPs of many human genes are located in different exons, such as exons 2 and 4, producing different antisense transcripts of neighboring genes [12]. The difference between the location of human and mouse L1-ASP may be a result of the evolution of these species [3]. L1 family members comprise numerous extracellular immunoglobulins and fibronectins, and the gene structure of human L1 family members differs from that of chicken and fruit fly, further revealing the different characteristics of ASPs among species.

#### 2.1.3. ASPs Are Located in Introns

Recent studies have shown that ASPs can be located in different gene introns; however, ASPs do appear to be preferentially located in the first intron, as observed for “initiator” (InR, the ASP of the human eukaryotic initiation factor 2α (*eIF-2*α) gene) [44], the mouse insulin-like growth factor 2 gene (*Igf2*) ASP [45] and pIRAIN, which generates an antisense transcript of insulin-like growth factor type I receptor (*IGF1R*) [46].

Some ASPs can also be located in the second intron, such as those of human BCL-2-related ovarian killer (*BOK*) [47], a human natural killer cell (NK) I type MHC receptor gene (killer cell immunoglobulin-like receptor surface, *KIR*) [48] and the murine *c-myc* gene [49].

ASPs can also be located in the third intron of a gene. For example, the ASP of the *PU.1* TF gene is located in intron 3 [50]. Of course, ASPs can be located in other introns. For instance, the ASP of the imprinted *Kcnq1* gene is located in intron 11 [51], and the ASP of the L1-COL11A1 (collagen type XI α1, *COL11A1*) transcript is located in the 46 intron. Previous studies have demonstrated that L1 ASPs can be distributed in various introns in different genes [12,38].

High-throughput sequencing has identified a great number of sense/antisense transcript pairs, yet only some of them exhibit a head-to-head formation, indicating that both directions of many genes fragments can generate transcripts [1,2,3]. Hence, we speculate that there are many ASPs located in the introns of other genes, though they remain to be identified and further studied.

#### 2.1.4. ASPs Are Located in the 3′-UTR

**Figure 3:**
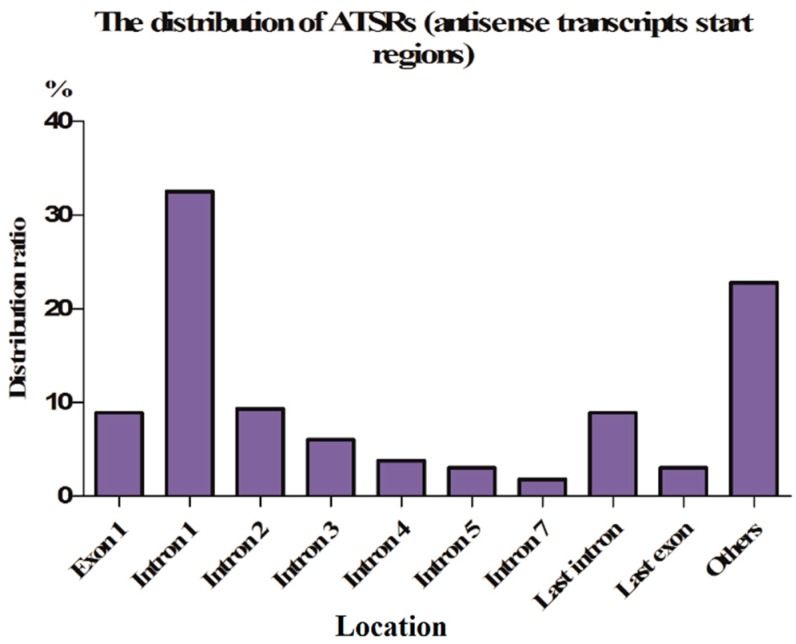
The distribution of antisense transcript start regions (ATSRs). Here, we hypothesized that one or more ASPs are located in ATSRs. The pie chart shows the ATSR distribution per genomic element. The distribution was evaluated for RefSeq genes with at least three exons for which it was possible to unambiguously distinguish the first and last introns. Globally, the distribution among 2671 RefSeq genes indicated that 94.3% of 2830 RefSeq genes contain an ATSR [33]. More than 50% of the ASPs are located in exon 1, intron 1 and intron 2.

To date, only rare ASPs have been found in the 3′-UTR of genes. The galanin (*GAL*) gene cluster, including *GAL1*, *GAL7* and *GAL10*, is a highly regulated galactose-induced genetic unit. *GAL7* and *GAL10* are tandem genes, and *GAL1* and *GAL10* are divergent genes; these three genes share a bidirectional promoter, and the *GAL10* antisense transcript is initiated within the *GAL10* 3′-UTR [31]. The antisense transcripts initiated by the ASP in the 3′-UTR influence the transcription initiation of sense transcripts by forming a tail-to-tail pair complement with them. Head-to-head sense/antisense transcript pairs are less common than those showing a tail-to-tail formation, likely because most antisense transcripts (especially those with a tail-to-tail formation) often play a role in post transcriptional regulation [52]. However, most ASPs are either located in the 5′-UTR, or in introns and exons (Figure 3) [33].

Although, the antisense transcripts of different human cell types correspond to exon, intron, promoter and terminator positions [53]. These antisense transcripts more frequently correspond to promoters and exons than they correspond to other positions of a gene. This finding coincides with the results of Finocchiaro *et al.* [33] who found that antisense transcript start regions (ATSRs) are more likely to be located in intron 1, followed by exon 1, intron 2 and the last intron of protein coding genes. Compared with other exons and introns, the TSSs of antisense transcripts demonstrate a strong tendency to be located in the first exon. Overall, more than half of ATSRs are located in exon 1, intron 1 and intron 2, indicating that ATSRs are distributed at the 5′-end of genes (Figure 3) [33].

### 2.2. ASPs Are Located in Adjacent Genes

According to the reported classification, natural antisense transcripts originating from an adjacent gene are considered trans-natural antisense transcripts [20,54]. Mammalian *Kcnq1to1* derives from intron 11 of the *Kcnq1* gene in the antisense direction and is only expressed paternally. In early embryonic development, Kcnq1to1 can silence three upstream genes, including cyclin-dependent kinase inhibitor 1C (*Cdkn1c*), solute carrier family 22 member 18 (*Slc22a18*) and Pleckstrin homology-like domain family A member 2 (*Phlda2*) [51], and the ASPs of these three genes are located in adjacent genes. Moreover, a number of L1 ASPs are located in the L1 5′-UTR (400–600 bp), and various antisense transcripts of adjacent genes originate from L1 ASPs if they are activated [12,36,37]. For example, in both tumor cells and normal tissues or cells, an L1-ASP with transcriptional activity has been shown to be responsible for the transcription of multiple contiguous genes [36,37].

### 2.3. ASPs Are Located in Intergenic Regions

ASPs located in intergenic regions may serve as alternative promoters or bidirectional promoters that share or compete for upstream regulatory elements (UREs) in the opposite direction [55]. It is worth noting that the promoters of the human immune deficiency virus type 1 (*HIV-1*) and human T-cell leukemia virus (*HTLV-1*) genes are bidirectional promoters [56,57], whereas the L1-ASP is an alternative promoter [36,37]. Mammalian L1-ASPs are tissue specific, and when this type of ASP is activated, a variety of antisense transcripts of different adjacent genes are initiated [12,36,37]. Unexpectedly, a bidirectional promoter can play a crucial role in the expression of related genes. For example, KIR3DL1, the bidirectional promoter of the human *KIR* gene, can regulate the frequent expression of KIR [48].

Regardless, the mechanism underlying the transcriptional control of bidirectional genes by this type of promoter remains largely unknown.

## 3. Characteristics of ASPs

Promoters can be recognized by RNA polymerase either directly or indirectly. The core promoter (CP), which is approximately 100 bp and situated near the TSS, is identified by and combined with other cis elements, such as enhancers and silencers, for transcriptional regulation [58]. CP elements identified in eukaryotic protein-coding genes include the TATAAA sequence (TATA box), upstream promoter element (UPE), TFII B recognition element (BRE), initiator and downstream promoter element (DPE), and the newly discovered motif ten element (MTE) near the DPE [58,59]. Bidirectional promoters contain a CCAAT box and CpG islands (CpGIs) (Table 1) [7].

### 3.1. ASPs Are RNA Pol II Promoters

Some of the identified ASPs are RNA polymerase (Pol) II promoters, which are recognized by and recruit RNA Pol II, including the ASPs of the murine *c-myc* gene [49,60], *α1(I) collagen* gene [61], and rat L1 [37]. A recent study confirmed that RNA Pol II participates in the initiation of antisense transcript transcription and explained how RNA Pol II associates with target genes and antisense transcripts. This study also showed that the *GAL10* ASP depends on transcription factor II D (TFIID), which recruits the Reb1p activating factor and interacts with the ASP at the TSS to produce antisense transcripts [31].

### 3.2. A TATA Box Is Not a Necessary Component of ASPs

The RNA Pol II promoter mainly integrates four components, a TATA box, an initiator, a GC box, and a CCAAT box. Although the first two components are also known as the CP [7], not all promoters contain a TATA box. For example, Trinklein *et al.* [62] reported that many bidirectional promoters lack a TATA box, but include a GC-rich box. In the human genome, only 10%–20% of promoters contain a TATA box, and GC boxes (6–8 nucleotides) are mainly concentrated in the TSS + 100 bp region [63]. However, the frequency of GC boxes in the chicken genome is only approximately 10% of that observed in humans; in the former, a GC content of approximately 0–70% occurs in the region from −600 bp to the TSS but is as high as 70% in the area neighboring the TSS. However, the CG content declines markedly due to the presence of TATA boxes from −25 to −30 bp [64].

Although the frequency of TATA boxes in eukaryotes is less than 20%, many promoter regions are TATA-less. For example, the *c-myc* gene ASP lacks a TATA box, has a low GC content, and contains ATCCAAAT sequences in the upstream TSS and a similar ATGCAAAT octamer binding site. Octamer binding sites also exist in the ASP of the *N-myc* (v-myc avian myelocytomatosis viral oncogene neuroblastoma derived homolog, Mycn) gene [49]. The bidirectional transcriptional promoters of the *HIV-1* and *HTLV-1* genes also lack TATA boxes but contain a GC-box, which is easily identified by and binds with other TFs [55,56].

A comparative analysis of sequence motifs showed that unidirectional promoters lack TATA boxes [43], and the frequency of TATA-less ASPs is higher than for SPs [7] (Table 1). It is noteworthy that some promoters without TATA boxes contain multiple TSSs [35]. For example, the rat L1 ASP lacks a TATA box, and different tissues, including the testicles, kidney and cerebellum, possess different TSSs [37]. However, some ASPs do harbor a TATA box, such as that of the *N-myc* gene [50,61] and the *Igf2r* (Igf2 receptor) ASP Air [65]. These findings indicated that TATA boxes are not necessary components of ASPs. A comprehensive prediction combined with experimentation would be beneficial for elucidating many types of promoters and their functions.

**Table 1:** Information of different transcription factors (TFs) in uni- and bi-directional promoters.

TATA-Less or TATA-Containing Promoters	TF Name	Location	Sequence (5′–3′)	Bidirectional Promoter	Unidirectional Promoter	Mechanism
TATA-box lacking promoters	INR	−3–+5	YYANWYY	25.30% [7,59]	30.80% [7,59]	It can function independently, and together with the TATA box or DPE [7,44,45,59].
	BRE	−37–−32	SSRCGCC	16.50% [7,59]	11.10% [7,59]	TFIIB–BRE interaction may play a dominant role in the assembly of the pre-initiation complex and transcription initiation [7,58,59].
	CCAAT	−75–−80	GGCCAATCT	12.9% [7,59]	6.90% [7,59]	Enhances the transcriptional rate [7].
	DPE	+28–+32	DSWYVY(T)	46.65% [7,59]	50.60% [7,59]	It can be recognized by TBP-associated factors (TAFs), such as TAF6 and TAF9 [58,59].
TATA-box containing promoters	Sp1	Ubiquitously located near the TSS [66]	GGGGCGG	/	/	Sp1 acts through its zinc finger domain at the carboxyl end to interact with GC rich sequences of downstream target genes [56,57,67]. It binds to the GC-rich box and recruits TFIID to regulate the bidirectional promoter HIV-1 LTR [66,68]. Recognizes the core sequence 5-GGGGCGGG-3 of the target promoter.
	NF-κB	/	/	/	/	It binds to the DNA sequence: 5-GGGRNYYY-3 [68,69,70].
	TATA	−25–−30	TATAAA	9% [59]	29% [59]	TATA box and INR interact synergistically when they are separated by 25–30 bp [7,44,59,64].
	CpGI	5′-regions of housekeeping genes and many tissues-specific genes [66]	size: 0.5–2 kb in length	77% [7,71], enriched binding sites of many transcription factors: Sp1, GABPA, MYC, E2F1, E2F4, Nrf1, YY1, NF-Y [42,72,73]; 86.37% [72].	56% [43]; 38%, compared with a bidirectional promoter, more lack GC-pairs [7,71]; 28.48% [72].	Hypermethylation of a CpGI in the promoter region usually suppresses gene expression [74,75,76], and the promoters of some tumor suppressor genes are hypermethylated in cancer [7,71,77]. Bi-directional genes tend to relate to housekeeping functions in metabolism pathways and nuclear processes [66,72].

Information derived from Orekhova and Rubtsov (2013) [7], Lepoivre *et al.* (2013) [42], Hildebrandt *et al*. (1992) [43], Silverman *et al.* (1992) [44], Duart-Garcia and Braunschweig (2014) [45], Gazon *et al.* (2012) [56], Yoshida *et al.* (2008) [57], Xie *et al.* (2007) [58], Yang and Elnitski (2008) [59], Abe and Gemmell (2014) [64], Wierstra (2008) [66], Zanotto *et al.* (2008) [67], Arpin-André *et al.* (2014) [68], Bentley *et al.* (2004) [69], Kobayashi-Ishihara *et al.* (2012) [70], Tufarelli *et al.* (2013) [71], Liu *et al.* (2011) [72], Lin *et al.* (2007) [73], Li *et al.* (2011) [74], Weber *et al.* (2010) [75], Rao *et al.* (2013) [76] and Shu *et al.* (2006) [77]. (DNA fragment no less than 500 bp with a GC-content ≥55% and Obs/Exp value ≥0.60). R, N, and Y denote for any purine, any pyrimidine, and any nucleotide, respectively.

### 3.3. ASPs Contain Specific TF Binding Sites (TFBSs)

ASPs contain many specific TFBSs that have been shown to respond *in vitro* to extracellular “stress”. For instance, bidirectional promoters in the human and rat genomes contain NF-Y TFBSs, which must combine with a CCAAT box to initiate gene transcription [67]. The PU.1 TF is a member of the hematopoietic cell line-specific ETS family and is necessary in normal cells [78]. Furthermore, the expression level of *PU.1* plays a key role in the fate of certain cells, and a slight decrease in its expression can lead to leukemia and lymphoma [79,80]. Previous studies have shown that the expression level of the *PU.1* gene is regulated through its proximal promoter [81] and a URE [80,82]. It has also been reported that *PU.1* can be regulated by antisense transcripts at the translational level and that UREs are essential for the generation of both antisense and sense transcripts [50]. UREs can be selected and shared by SPs and ASPs. KIR3DL1, the promoter of the human *KIR* gene, is a bidirectional promoter with a myeloid zinc finger 1 (MZF-1) binding site in the CP region [48].

In mammalian genomes, many sense/antisense transcript pairs are driven by bidirectional promoters. A comparative analysis showed that the composition of CP elements between unidirectional and bidirectional promoters differs, including the four main components. In addition, protein-coding genes share RNA Pol II, whereas non-protein encoding genes share RNA Pol III [7]. The different types of TFs found in different promoters and their ratios are listed in Table 1.

Many studies have shown that certain ASPs belong to TF-dependent promoters. For example, the *GAL10* ASP is TFIID and Reb1p dependent [31]. Lyle *et al.* [67] reported that Air promoter regions also harbor specific TFBSs, such as AP2, MseI and Sp1. Additionally, more than one-third of *c-myc* promoters must recruit the TF P-TEFb to activate transcriptional activity in mouse embryonic stem cells (mESCs) [60,83]. The different L1 splicing forms [36,37] mentioned above are related to L1 ASP regulation by TFs [43]; Lepoivre *et al.* [42] identified binding sites for GATA3, ETS1 and RUNX1 TFs, which contribute to cell differentiation and development, in the L1 ASP. Bidirectional promoters are more frequently combined with other TF binding sites, such as NRF-1, CCAAT, YY1 and ACTACAnnTCCC; however, few TFs show a preference for participating in unidirectional transcriptional promoters [72,73], including MYC, ELK1, NF-Y, SP1, ATF, GABPA, SREB-1, NF-E2 and SOX, STAT5A, and NF-1-9. We suggest that antisense transcripts could be transcribed in a temporally regulated, tissue-specific expression pattern, as these specific TFBSs within ASPs result in specific temporal and spatial activity patterns.

### 3.4. ASPs Are Selected by Evolution and Possess Lower Activity than SPs

Previous studies have indicated that approximately 20% of the human genome can produce nature antisense transcripts [1,53]; in contrast, more than 70% of the mouse genome produces antisense transcripts [3]. In vertebrates, many new functional transcripts and at least some coding functional transcripts in the opposite direction have arisen during evolution through functional selection. It was shown that the emergence of bidirectional promoters is conducive to maintaining the specificity of different species [41,84]. A case in point is the location of the mouse L1 ASP, which unlike that in humans (Figure 2), is the result of evolutionary selection [3].

Additionally, a large number of experimental investigations have shown that numerous pairs of sense/antisense transcripts exist in the human genome, even though the expression level of antisense transcripts is lower than that of sense transcripts [7,33]. We speculate that this phenomenon is due to the following reasons. First, ASPs have a higher CG content, resulting in an elevated level of ASP methylation and, thus, inhibition of their transcriptional activity; Second, ASPs exhibit higher activity in the embryonic period than in later stages, as most antisense transcripts play an important role during early growth and development; Third, many factors regulate the activity of ASPs; Fourth, an antisense transcript may be less stable compared with sense RNA, exhibiting a shorter half-life; Fifth, in the sense direction, a more active U1 snRNP protects the sense transcripts of protein-coding genes from premature cleavage and polyadenylation in promoter-proximal regions, thus reinforcing the transcriptional directionality of genes [85]; Sixth, in some cases, antisense transcripts can directly or indirectly bind to the SP region of their target genes, allowing for an open chromatin conformation and positively regulating the expression of sense transcripts. As the expression of a large number of antisense transcripts is very low and displays a temporally and spatially specific pattern, the transcriptional activity of the corresponding ASPs should also present a temporal and spatial expression pattern under the influence of different TFs. There are, however, exceptions. For example, for the *hTPH2* 5′-UTR bidirectional transcriptional promoter, ASP activity is much stronger than that of the SP [40].

## 4. Factors Affecting the Activity of ASPs

As ASPs are distributed throughout the genome, they can be activated by many types of “signals” to generate antisense transcripts. DNA unwinding requires the binding of one or more TFs to corresponding sites in ASPs to simultaneously interact with the template chain and form a transcription initiation complex; the ASP is then activated to drive antisense transcription [31].

### 4.1. The Activity of ASPs Is Closely Related to the Functions of Different Genes

The activity of ASPs is strongly related to the functions of different genes. Genes expressed during embryonic development are induced and transcribed quickly when cells are proliferating. When the initiation of antisense transcription is complete, the activity of ASPs declines rapidly or is even silenced, though it can be activated again at a later time. Thus, there are ASPs with temporal specificity or spatial regularity, such as the *c-myc* [50] and *N-myc* genes [61]. In 1992, Silverman *et al.* [44] found that the expression of *eIF-2α* increased rapidly in G0 phase, whereas both the eIF-2α sense transcript and the InR antisense transcript showed very low levels in G1 phase, demonstrating that eIF-2α/InR exhibits time-dependent expression. In another study, approximately 60% of long noncoding RNAs (lncRNAs) in ESCs were shown to be located near the TSS of coding genes. When the L1 ASP is responsible for adjacent gene transcription, natural antisense transcripts regulate cell differentiation and development by combining with matching mRNAs to form dsRNAs [43]. Multiple such genes are present in cells, guaranteeing the production of sufficient antisense transcripts together with sense transcripts for the regulation of an individual’s early (embryonic) cell differentiation and development [11].

For oncogenes such as the L1 ASP, specific natural antisense transcripts of cancer genes can be produced [37,71]. Additionally, approximately 25% of promoters in cancer cells are bidirectional transcription promoters with CpGIs and are highly methylated [77]. When cancer cells proliferate, the activity of ASPs is relatively strengthened [71].

It was shown that the expression of tissue-specific promoter-associated ncRNAs (pancRNAs) is positively correlated with the expression of mRNAs and that pancRNAs can activate facultative genes when constitutively expressed genes lack pancRNAs [86]. That is to say that the ASPs of facultative genes show strong activity and the ASPs of constitutively expressed genes show no or weak transcriptional activity.

### 4.2. ASP Activity Is Associated with the Distribution Density of TSSs

The methylation signals of the 5′-end of intron 1 and the promoter region in most human genes are very low. However, TSS signals of antisense transcripts are gradually reduced from the 5′-end to the 3′-end of intron 1 but are markedly higher than those of the other introns, by 1.5–5 times, and the antisense transcript TSSs are normally distributed on both sides of the junction between exon 1 and intron 1 [33]. In other words, antisense transcript TSSs are mainly distributed at the 3′-end of exon 1 and the 5′-end of intron 1. He *et al.* [53] reported the interesting finding that higher ASP activity is found with a greater distribution of antisense transcript TSSs.

Cap analysis of gene expression (CAGE) in the human genome showed that a transcriptional unit possesses 16.3 sense transcript TSSs and 5.8 antisense transcript TSSs [87,88]. Conley *et al.* [87] found that partial sense transcript TSSs originate from the same alternative promoter as the mRNA-TSSs previously reported by Carninci *et al.* [88]. Interestingly, Osato *et al.* [89] argued that sense transcript TSSs are the result of transcriptional collision between antisense and sense transcripts.

### 4.3. ASP Activity Is Associated with the Degree of Methylation of CpGIs

Promoter regions are demethylated *in vivo*, decreasing their methylation signals compared to those of other areas [67,90]. In the vertebrate genome, an interesting observation is that genes are expressed in multiple tissues if their promoters contain CpGIs but are only active in a particular pattern when ASPs lack CpGIs [63]. This finding suggests that the temporal and spatial expression of antisense transcripts in particular tissues might be induced by the extent of CpGI methylation in their ASPs.

In fact, the lower the CpGI methylation signals in an ASP, the denser is the distribution of the antisense transcript TSS signal, and the greater is the activity of the ASP. More than 90% of bidirectional promoters contain significant CpGIs and CCG and CGG repeat sequences, which are enriched in the upstream and downstream regions of the TSSs of bidirectional promoters [77,91]. In contrast, only 56% of unidirectional transcription promoters contain CpGIs [43]. Bidirectional promoters in mESCs harbor many CpGIs, with an asymmetric distribution in the upstream and downstream regions of TSSs, which are inhibited by polycomb complexes [43].

The methylation level of CpGIs is negatively related to gene expression levels, with an inhibitory effect on gene transcription [74,75]. Weber *et al.* [75] revealed that the L1-cMet ASP is highly methylated in most human cells: when the ASP is demethylated, L1 antisense transcript expression is remarkably increased, whereas cMet expression and methylation signals are reduced.

In the chicken genome, most CpGIs are maintained in a state of demethylation [74], and the GC content of the 5′-UTR is positively related to the gene expression level, the breadth of expression and maximum expression, demonstrating that the GC content plays a key role in chicken gene regulation networks [76], though further analysis is necessary to elucidate the molecular mechanism.

### 4.4. ASP Promoter Activity Is Strongly Regulated by TFs

In the complex genome, a promoter is a characteristic sequence showing a transcriptional function and cis regulation, located upstream of a TSS, and contains a variety of sequence motifs involved in gene regulation [63]. Eukaryotic promoter regions contain motifs responsible for the transcription of the gene, and core elements often coexist and form composite motifs, including TFBSs, short tandem repeats, G-quadruplexes, and CpGIs.

Previous studies have shown that TFs play key roles in regulating the activity of ASPs. For example, Sp1 binding sites play an important role in regulating the activity of ASPs, such as by participating in HTLV-1 ASP transcription [56,57], combining with the GC box and recruiting the TF TFIID to activate the transcription of antisense transcripts [66]. Various studies have shown that NF-κB is also involved in ASP regulation [69,70]. Moreover, the HIV-1 long terminal repeat (LTR) acts as a bidirectional transcriptional promoter, the activity of which is regulated by NF-κB- and Sp1 binding sites in both orientations [68]. Of course, ASPs are regulated by specific TFs determined by the TFBSs present; these TFs can be independent or play a role in complex formation with RNA II to activate the ASP.

It is worth noting that one TF can interact with different gene promoters and regulate the expression of different genes. The human double homeobox 4 (*DUX4)* gene encodes the TF DUX4, which is expressed in testicular germ cells but is epigenetically silenced in ovarian tissue. When this TF binds to the ASPs of the *MLT1C*, *THE1C* and *DDX10* genes, lncRNAs and natural antisense transcripts can be produced [92].

### 4.5. The U1 Small Nuclear Ribonucleoprotein (U1 snRNP) Affects ASP Promoter Activity

The mammalian U1 snRNP exhibits a weak recognition capability for natural antisense transcripts but strong recognition of sense transcripts [85]. Correlation analysis of a mathematical model of the relationship between sense transcripts or antisense transcripts and U1 showed that with an increase in the age of genes, the U1 site in downstream sense regions at the 5′-end (the first 1 kb) of protein-coding genes is significantly positively correlated with sense transcripts and negatively correlated with antisense transcripts. Interestingly, the U1 site in upstream antisense regions at the 5′-end (the first 1 kb) of protein-coding genes shows a significant negative correlation with sense transcripts and a positive correlation with antisense transcripts, suggesting that at least a subset of uaRNAs may be functionally important for antisense transcripts to become more extensively transcribed. Sense U1 sites are intensively distributed within the TSS + 200 bp region [85]. These observations indirectly demonstrate that the U1 snRNP restrains the activity of ASPs but enhances the activity of SPs.

### 4.6. Polyadenylation Site Signals Are Implicated in ASP Activity

It has been shown that chicken *GHR* mRNA transcripts are initiated from at least two SPs (V1 and V6), with a polymerase phosphorylation signal (PAS) in intron 6 [36]. Are ASPs also affected by PASs? Almada *et al.* [85] discovered that the percentage of mammalian PASs in the upstream region of TSSs was twice as high as in downstream regions. In addition, correlation analysis of a mathematical model of the relationship between sense/antisense transcripts and PASs revealed that the expression of both sense and natural antisense transcripts is significantly negatively correlated with both upstream and downstream PASs [85]. When ASPs are subject to the inhibitory effect of the U1 snRNP and PASs, the expression of natural antisense transcripts is much lower than that of sense transcripts. Compared with the transcription of mRNA, the extension of antisense transcripts is subject to relatively more interference from PASs, and transcription is forced to terminate more quickly [85]. PASs clearly also affect the activity of ASPs. Most genes exhibit more than one antisense transcript, and different antisense transcripts may be transcribed by one or more ASP. The formation of different transcripts is also highly dependent on PASs.

Some other enzymes may influence the activity of ASPs. Early in 1998, Vanhée-Brossoll and Vaquero reported a negative correlation between *α1 (I) collagen* gene mRNA and natural antisense transcripts [93]. In addition, the activity of the *eIF-2α* ASP may be regulated by an InR-associated binding protein through the control of RNA Pol II to interact with the InR-ASP. When entering the cell cycle, the expression of InR is low because its association with Inr-associated binding protein is restrained [45]. Moreover, topology isomerase I interacts with TF sites within the ASP, playing an indispensable role in stimulating and regulating the activity of this promoter [45]. The structure of bidirectional promoters is associated with lncRNAs, which are induced or inhibited in a rapid retinoic acid-dependent manner. The rat L1-ASP is Dicer enzyme dependent [37]. This process is similar to the generation of miRNAs, as it is subject to dsRNA formation between sense and antisense transcripts and then produces small interfering RNAs (siRNAs) or miRNAs through Dicer cleavage [94,95]. However, it remains to be determined whether certain enzymes in tissues or cells control the activity of ASPs. If the genes encoding these enzymes show tissue specificity, the regulation of the ASP will also be tissue specific, ultimately resulting in natural antisense transcripts only being expressed in some tissues.

### 4.7. Stimulation by Material Outside of Cells Influences the Activity of ASPs

The cell environment is particularly important for cell survival and reproduction, and many complex molecular biological processes, such as DNA replication, RNA transcription and protein translation, must be carried out in this environment. However, extracellular stressors may interfere with the transfer of genetic information. In studying the impact of stimuli on the cell environment, most researchers are currently focusing on extracellular inhibitors.

The expression of the *α2 (I) collagen* gene in cartilage cells and its corresponding promoter inactivates and decreases the expression of *α2 (I) collagen* to one-third of initial levels after BrdU treatment [62]. Through bioinformatics analysis, Nigumann *et al.* [39] revealed that L1-ASP could initiate the expression of natural antisense transcripts with high expression in expressed sequence tag databases. Recent studies have shown that various cytosine nucleosides, such as azacytidine, cytidine analogues, and DNMT inhibitors, can activate illegitimate transcription from the L1-ASP [44]. Weber *et al.* [75] performed an in-depth analysis of the regulation of L1-cMet ASPs and found that most human L1-cMet ASPs are hypermethylated, significantly increasing the expression of L1 antisense transcripts. Additionally, L1-cMet transcript expression levels and methylation signals are reduced. The same result can be obtained using micromolar doses of decitabine in cancer cell cultures.

### 4.8. ASP Activity Is Influenced by Chromatin and Histone Modifications

Epigenetic regulatory pathways influence gene transcription by modulating chromatin structure without altering primary DNA sequences [96]. The mechanisms that regulate chromatin structure include ATP-dependent chromatin remodeling, replacement of major histones with variants, cytosine methylation, the small RNA pathway, and covalent posttranslational modifications of histones [97]. Histone H3 trimethylated at Lys-4 and Lys-36 (H3K4met3, H3K36met3) can be used as a marker of chromatin activity, whereas histone H3 trimethylated at Lys-9 and Lys-27 (H3K9met3, H3K27met3) is a marker of transcriptionally silent chromatin. Histone acetylation, such as H3 acetylated at Lys-14 (H3K14ac), can also alter chromatin conformation, allowing transcriptional co-activating factors access to the promoter region and consequently favoring transcription. These markers associated with antisense promoters are distinct from the features associated with sense transcription. In general, high levels of antisense transcription result in pronounced differences in a broad range of chromatin features, both for the sense promoter and within the gene body. Features associated with newly deposited dynamic chromatin-histone H3 acetylation, turnover, chromatin remodeling enzymes and histone chaperones are typically increased, whereas features associated with established chromatin-H3K79me3, H3K36me3 and H2B123ub are reduced. These features are distinct from those associated with sense transcription [98]. Additionally, ASPs can form an R-loop structure which can be disfavored *in vitro* and *in vivo* by ribonuclease H1 overexpression, thereby resulting in gene down-regulation [34]. Overall, ASPs can regulate themselves at a low-affinity chromatin structure, and some antisense transcript can recruit other TFs to bind to the SP region to increase chromatin accessibility, followed by a high level of sense transcript expression [26].

Regulation of the activity of ASPs is complicated, as there are many factors affecting ASPs’ activity, and the same ASP may be influenced by many factors. This situation can be exemplified by the fact that the activity of different mESC ASPs is P-TEFb dependent, in addition to being affected by CpGIs [54].

## 5. Conclusions

Briefly, ASPs can be located in any position within a gene or intergenic region. The position of ASPs determines the initiation of the antisense transcript, and the dsRNAs formed by antisense transcripts match their target sense transcripts, ultimately determining the role of antisense transcripts in regulating gene expression. Therefore, it is necessary to conduct further analyses of the locations of interesting ASP in target genes, which will provide beneficial clues for understanding the initiation of antisense transcripts.

The initiation of antisense transcription is an extremely complex process. Many transcriptional elements can combine with ASPs, with different elements playing different roles, and interactions among components may even exist. Subsequent efforts should focus on the interactions between these transcriptional elements to elucidate the molecular mechanisms of ASPs and their antisense transcripts.
